# In-bag dislocation of intraocular lens in patients with uveitis: a case series

**DOI:** 10.1186/s12348-015-0036-1

**Published:** 2015-04-02

**Authors:** Lingwei William Tao, Anthony Hall

**Affiliations:** Department of Ophthalmology, The Alfred, 55 Commercial Road, Melbourne, Victoria 3004 Australia; The Alfred, PO Box 315, Prahran, Victoria 3181 Australia

**Keywords:** Intraocular lens dislocation, Uveitis, Cataract surgery

## Abstract

**Background:**

Improvement in surgical devices and intraocular lenses has made modern cataract surgery a safe procedure with decreasing complication rates. Intraocular lens dislocation is a serious complication after cataract surgery. Although most dislocations occur during the first week postoperative period, late intraocular lens dislocation occurring 3 months or later post-surgery has been reported with increasing frequency in recent years as a result of progressive zonular dehiscence. We report the clinical features, management and outcomes of five cases of late in-bag dislocation of intraocular lens in patients with underlying uveitis. This is a retrospective case series and literature review.

**Results:**

We identified five eyes in five patients with uveitis and late in-bag intraocular lens dislocation. Two patients had multifocal choroiditis, two herpetic uveitis and retinitis and two Fuchs’ heterochromic iridocyclitis in five patients. Mean age at the time of cataract surgery was 50. Best vision ranged from counting fingers to 6/18 preoperatively and ranged from 6/36 to 6/6 postoperatively. All had right eye dislocation with mean time from initial cataract surgery to intraocular lens dislocation of 81 months. Explantation of dislocated intraocular lens and vitrectomy were performed in four cases; three had anterior chamber intraocular lens placement. One case was managed conservatively. Best vision ranged from light perception to 6/7.5 at time of dislocation and ranged from 6/36 to 6/6^−2^ at follow-up.

**Conclusions:**

Late in-bag dislocation intraocular lens can complicate cataract surgery in patients with underlying uveitis. This case series identified that the mean time to in-bag intraocular lens dislocation in five uveitis patients was 81 months after uncomplicated cataract surgery, comparable with the time reported in the available literature of patients with pseudoexfoliation syndrome. This series also found that lens explantation and replacement with anterior chamber intraocular lens achieved good outcomes. Further investigation is warranted to ascertain the strategies to identify patients at risk and to prevent and better manage intraocular lens dislocation in patients with uveitis.

## Background

Cataracts are known to develop in an accelerated rate in patients with uveitis [1]. Improvement in surgical techniques and intraocular lenses (IOLs) has made modern cataract surgery a safe procedure in patients with uveitis [2]. IOL dislocation is a serious complication after cataract surgery. Most IOL dislocations happen during the first week postoperative period. Late IOL dislocation is defined as occurring 3 months or later after cataract surgery. Late IOL dislocation has been reported with increasing frequency in recent years [3,4]. The rate of posterior chamber intraocular lens (PCIOL) dislocation ranges between 0.2% and 2.8% [5-7]. Described risk factors for in-bag dislocation include pseudoexfoliation, uveitis, trauma, vitrectomy and increased axial length [3,7-13]. In this case series, we report the clinical features, management and outcomes of five cases of late in-bag dislocation of PCIOL in patients with uveitis.

## Methods

A retrospective chart review was performed. Patients were identified from computerised coding of diagnosis and surgery. Five consecutive cases were collected from a single uveitis practice, and their clinical features, management and outcomes were then extracted from patients’ histories and operation notes. In this series, we presented the following patient data - age, gender, side of dislocation, past medical history, relevant ocular history, age at initial cataract surgery, best visual acuity preoperatively and postoperatively, comment of cataract surgery, type of lens implant, time to dislocation, date of reported dislocation, visual acuity when lens dislocated, management of dislocation, date of dislocated lens removal, best visual acuity post-repair and final uveitis status.

## Results

### Case 1

An 81-year-old Caucasian male presented with long-standing right-sided multifocal choroiditis and retinal vasculitis complicated by choroidal neovascularization with a macular scar and bilateral moderate glaucoma. He developed progressive anterior and posterior subcapsular cataract. Preoperatively, the patient’s uveitis was controlled with topical steroids only. He underwent uncomplicated phacoemulsification cataract extraction with a 19.0D Alcon SN60WF in-bag implant in 2000. Postoperatively, his vision was initially 6/36. Due to his choroidal neovascular membrane, the vision in his right eye progressively deteriorated to light perception only by 2009. He represented in 2013 at the age of 80, 165 months after his initial right eye IOL implant, with in-bag dislocation of his PCIOL. He was managed conservatively due to the poor visual potential.

### Case 2

A 49-year-old Caucasian male presented with a dense right-sided cortical cataract in 2007 at the age of 44. HIV infection was diagnosed in 1997. His ocular history included bilateral keratoconus, bilateral uveitis, and multifocal choroiditis, which had been treated with eight intravitreal triamcinolone (IVTA) injections previously and was quiescent at the time of his cataract surgery. His right eye vision was 3/60 preoperatively. He underwent uncomplicated phacoemulsification cataract surgery and implantation of a 15.5D Alcon SN60WF into the bag. Postoperatively, his acuity improved to 6/12. Subsequently, he underwent right eye vitrectomy for long-standing vitritis in 2008. Seventy-five months after his initial PCIOL implant, he represented with visual loss in the right eye due to an in-bag IOL dislocation. He underwent a pars plana vitrectomy and removal of IOL. He was left aphakic and managed with a hard contact lens. On the last review in June 2014, his right eye corrected visual acuity was 6/7.5.

### Case 3

A 64-year-old Caucasian female presented with right-sided cataract in 2009 at the age of 59. She had a background history of rheumatoid arthritis treated with methotrexate. She had developed right herpetic retinitis in 2005. She underwent uncomplicated phacoemulsification with an Alcon SN60WF inserted in-bag. Loose zonules were noted at the time of the cataract surgery, and no capsular tension ring was inserted. Postoperatively, her acuity was 6/6. In May 2013, 47 months post-surgery review, there was no evidence of IOL instability; however, she represented 6 months later in November 2013 with right eye fluctuating vision and a right-sided in-bag IOL dislocation. Two months later, she underwent a right-sided anterior approach PCIOL explantation and anterior vitrectomy with anterior chamber intraocular lens (ACIOL). Postoperatively, her corrected visual acuity was 6/12.

### Case 4

A 57-year-old Asian male had initial right eye cataract surgery in 1998 at the age of 40 in Singapore. Background history included asthma and allergic rhinitis. He had an ocular history of right-sided Fuchs’ heterochromic iridocyclitis and secondary glaucoma. In 2002, approximately 48 months after his right eye PCIOL implant, the patient developed intermittent right-sided ocular inflammation. On examination, his vision was 6/6 and was found to have an in-bag superonasally displaced PCIOL, rubbing against the posterior surface of the iris and causing inflammation. In 2013, the PCIOL dislocated inferiorly. The patient underwent right eye PCIOL removal with pars plana vitrectomy and removal of IOL with secondary ACIOL placement. Postoperatively, his acuity was 6/6^−2^.

### Case 5

A 45-year-old Caucasian male presented with right eye cataract secondary to previous bilateral uveitis in 2008 at the age of 39. Background history includes type 2 diabetes diagnosed in 2011. Ocular history included herpetic keratouveitis with bilateral acute retinal necrosis diagnosed in 2006 (now quiescent) and bilateral relapsing remitting macular oedema treated with multiple IVTA injections. His preoperative right eye vision was counting fingers, and he underwent an uncomplicated phacoemulsification with a 19.0D Alcon SN60WF PCIOL inserted into the bag. Postoperatively, the patient continued to have intermittent severe macular oedema and received three further right eye IVTA injections between 2008 and 2013. His right eye vision has improved to 6/9 by February 2013; however, some right eye PCIOL dislocation was noted. Sixty-four months after his initial PCIOL implant, the patient was found to have right eye in-bag PCIOL dislocation and a vision of 6/24^−1^ in June 2013. Two months later, he underwent a right-sided anterior approach IOL extraction and secondary anterior chamber IOL implantation. On last review, his right eye vision was 6/36.

## Discussion

We described five late in-bag PCIOL dislocations in patients with underlying uveitis. Tables 1 and 2 summarised their key clinical features, management and subsequent outcomes. The age of patients at the time of cataract operation ranged from 39 to 67 years (mean: 50 years; median: 44 years). Four out of the five patients were male. There were two cases of multifocal choroiditis, two treated herpetic viral retinitis and uveitis, and two Fuchs’ heterochromic iridocyclitis which were also treated with IVTA injections. All patients had in-bag dislocation of PCIOL in their right eyes. The best vision ranged from counting fingers to 6/18 preoperatively and postoperatively varied from 6/36 to 6/6. There were four Alcon SN60WF PCIOLs, and it was unknown for case 4. Circular capsulorhexis and divide-and-conquer phacoemulsification technique was performed in four eyes, and in case 4, the surgical technique was not known. Case 2 had a dense cortical cataract, and case 3 was found to have a loose capsular bag intraoperatively. Case 4 had intermittent inflammation, and case 5 had severe macular oedema postoperatively. Both case 2 and case 5 received multiple IVTA injections. In this retrospective series, there was no information on the cataract density or the duration of the cataract surgeries; however, those patients were generally younger and had less dense cataracts compared to age-related cataract patients, and they also require lower power IOLs.

**Table 1:** **Summary of Cases**

**Case number**	**Eye/age/sex**	**Past medical history**	**Ocular history**	**Uveitis diagnosis**	**Date of cataract surgery**	**Best VA preop**	**Best VA postop**	**Comment of surgery**	**Type of IOL**	**Time to dislocation (months)**	**Date of reported dislocation**	**VA when lens dislocated**	**Management of dislocation**	**Date of IOL removal**	**Best VA post repair**	**Last review**	**Final uveitis status**
1	R/81/M	Hypertension, osteoarthritis, ischaemic heart disease, dyslipidaemia	RE CNV macular scar, bilateral moderate glaucoma, RE PKE/IOL, LE PKE/IOL	RE multifocal choroiditis and retinal vasculitis	10 February 2000	VAR 6/24	VAR 6/36	Uncomplicated	PCIOL 19.0D SN60WF	165	28 November 2013	VAR perception of light	Conservative management		VAR perception of light	09 May 2014	RE multifocal choroiditis and retinal vasculitis
2	R/49/M	HIV positive (good CD4 count)	Bilateral keratoconus, RE Fuchs’ heterochromic iridocyclitis, pseudophakia, quiescent bilateral multifocal choroiditis quiescent, persistent old right vitritis	Bilateral multifocal choroiditis, RE vitritis	06 September 2007	VAR 3/60	VAR 6/12	Dense cortical cataract	PCIOL 15.5D SN60WF	75	13 December 2013	VAR 6/36	IOL removed via AC and a corneal section	16 December 2013	VAR 6/7.5 with hard contact lens	24 June 2014	Bilateral multifocal choroiditis, RE vitritis
3	R/64/F	Rheumatoid arthritis (1995)	RE acute retinal necrosis (2005)	Right acute retinal necrosis secondary to herpetic retinitis (2005)	11 June 2009	VAR 6/18	VAR 6/6	Very loose bag	PCIOL 17.5D SN60WF	53	28 November 2013	VAR 6/7.5	PCIOL explantation and ACIOL placement	30 January 2014	VAR 6/12	27 April 2014	RE herpetic retinitis
4	R/56/M	Hay fever, asthma	Bilateral steroid-induced glaucoma, RE Fuchs’ heterochromic iridocyclitis, RE inferiorly dislocated IOL, RE vitrectomy and PKE/IOL 14/11/13 MTA4UO +14	Presumed Fuchs’ heterochromic iridocyclitis secondary to inferiorly dislocated PC IOL RE	1998		VAR 6/6	Intermittent inflammation		48	05 August 2002	VAR 1/60	PCIOL explantation with vitrectomy, ACIOL placement	14 November 2013	VAR 6/6 - 2	15 May 2014	RE Fuchs’ heterochromic iridocyclitis
5	R/45/M	Type 2 diabetes (2011)	Herpetic keratouvetitis, bilateral retinal necrosis (2006), bilateral relapsing remitting macular oedema, vitritis, multiple IVTA	Herpetic keratouvetitis, bilateral retinal necrosis (2006	07 February 2008	VAR counting finger	VAR 6/9	Bilateral severe macula oedema, initially treated by orbital floor steroids, failed to respond, later responded well to multiple IVTA (16/05/2008 to 27/06/2008)	PCIOL 19.0D SN60WF	64	21 June 2013	VAR: 6/24 - 1	PCIOL explantation with anterior vitrectomy, IVTA and ACIOL placement	28 August 2013	VAR 6/38	22 April 2014	Bilateral retinal necrosis

AC, anterior chamber; ACIOL, anterior chamber intraocular lens; CNV, choroidal neovascularisation; HIV, human immunodeficiency virus; IOL, intraocular lens; IVTA, intravitreal injection of triamcinolone; LE, left eye; PCIOL, posterior chamber intraocular lens; PKE, phacoemulsification; postop, postoperatively; preop, preoperatively; RE, right eye; VA, visual acuity; VAR, visual acuity right eye.

**Table 2:** **Analysis of Cases**

**Case number**	**Age**	**Mean age**	**Median age**	**Std dev age**	**Date of cataract surgery**	**Date of reported dislocation**	**Time to dislocation (months)**	**Mean time to dislocation (months)**	**Median time to dislocation (months)**	**Std dev (months)**	**Date of IOL removal**	**Time between dislocation and removal of IOL**	**Last review**	**Follow-up (months)**	**Age at time of surgery**	**Mean age**	**Median age**	**Std dev age**
1	81	59	56	14.27	10 February 2000	28 November 2013	165	81	64	48.10			09 May 2014	6	67	49.8	44	12.52
2	49				6 September 2007	13 December 2013	75				16 December 2013	3 days	24 June 2014	6	44			
3	64				11 June 2009	28 November 2013	53				30 January 2014	63 days	22 April 2014	5	59			
4	56				1998	5 August 2002	48				14 November 2013	11 years and 3 months	15 May 2014	6	40			
5	45				07 February 2008	21 June 2013	64				28 August 2013	2 months and 1 week	22 April 2014	6	39			

Std dev, standard deviation.

The time from initial cataract surgery to in-bag dislocation of PCIOL ranged from 48 to 165 months (mean: 81 months and median: 64 months). This is similar to the time frame reported by the largest series of pseudoexfoliation patients - mean 86 months, range 57 to 115 months [11]. The vision at time of dislocation ranged from light perception to 6/7.5. Case 1 with vision of light perception was managed conservatively due to his poor prognosis, and the remaining four cases had additional surgeries. The surgical approach to the dislocated IOL consisted of pars plana vitrectomy with secondary ACIOL in one eye, pars plana vitrectomy with no secondary ACIOL in one eye, and anterior approach lens explantation and secondary ACIOL in two eyes. The explanted IOLs were not sent for histology, and case 4 was found to have a macroscopically fibrosed lens capsule. The final vision after the additional surgeries ranged from 6/38 to 6/6^−2^. At 6-month follow-up, half of the patients had improved vision and were comparable to their best vision post-initial cataract surgery - case 2 final vision was 6/7.5, better than 6/12 post-cataract surgery; and case 4 final vision was 6/6^−2^, similar to 6/6 post-cataract surgery. Of the half with worsening vision at 6-month follow-up, case 3 vision was 6/12 compared to 6/6 post-cataract surgery, but was better than pre-cataract vision of 6/18; case 5 follow-up vision was 6/36 compared to 6/9 post-cataract surgery, but was better than pre-cataract vision of counting fingers. Overall, final visual outcomes of the five cases at 6-month review were equal or better than their pre-cataract vision. This is consistent with the available literature on described cases with in-bag IOL dislocation, which suggested that prognosis of eyes with this syndrome with any method of management is quite good with most eyes regain their preoperative vision [3].

The exact incidence of late in-bag IOL dislocation following uneventful cataract surgery is unknown. As our series included patients who had their initial surgery elsewhere, we are not able to provide a denominator or a rate of late in-bag IOL dislocation. The cause of in-bag IOL dislocation is thought to be multifactorial, and 90% of reviewed cases are associated with predisposing factors - pseudoexfoliation, uveitis, trauma, vitrectomy and increased axial length [9,10,12]. There are separate reports of intracapsular dislocation of crystalline lens in uveitis patients [14,15]. In an experimental rabbit model, inflammation of the ciliary body leads to fibrin and leukocyte deposition which destabilised the zonular insertion sites [16]. In addition, several reported cases identified sphincter effect of capsular fibrosis causing contraction around an intact capsulorhexis [11] which could occur as early as 3 months post-cataract surgery, and profound shrinkage was reported in cases with uveitis [9]. It was suspected that IVTA injections and topical steroid use could weaken the zonules, as two of the five reported cases had multiple IVTA injections. However, there is limited evidence in the available literature to substantiate such theory nor there has been reported in the diabetic maculopathy cohort requiring multiple IVTA injections to have an increased rate of capsular dislocation. We postulate that in uveitis patients, the underlying inflammation would and the subsequent treatment with topical or intravitreal steroid may weaken the zonules; in addition, the younger age at the time of initial cataract surgery and the uveitis may likely contribute to profound capsular shrinkage. The centripetal force exerted by the capsular contraction adds further strain to the already weakened zonules and results in premature failure and dehiscence of the suspensory apparatus.

Several preventative measures have been described in the literature - an appropriately sized capsulorhexis or the use of capsular tension ring could reduce capsular contraction, therefore delaying the onset of IOL dislocation; tailored phacoemulsification could preserve zonular integrity by using chopping techniques and aspiration of cortex directed in a tangential orientation rather than perpendicular to the zonules [17]. Some literature also suggests that surgeons must look for phacodonesis and in cases of clinically evident zonular instability, to consider the possibility of a primary sutured IOL or endocapsular, scleral-fixated tension rings [8]. In this retrospective case series, preoperative phacodonesis and postoperative IOL stability was not documented in all cases, and this may warrant further study to compare these clinical findings between uveitis-induced and age-related cataract patients.

The management of dislocated IOLs depends on clinical features and surgeon’s preference. Current literatures have described several methods when approaching management of dislocated IOLs. Oshika et al. described a simple fixation technique using a double-armed polypropylene suture passing one over and one under the haptic and out through the sulcus via a corneal stab incision [18]. An alternative method of fixation of the IOL to the iris with modified McCannel suturing has also been considered [19,20]. Although iris and ciliary body fixation of subluxated IOLs has the theoretical advantage of moving the IOL out of the anterior chamber, there is still significant uveal contact. In addition, pars plana approach is the only technique available for explantation of IOLs dislocated significantly posteriorly into the vitreous cavity. There is insufficient evidence to support the superiority of sclera- or iris-sutured PCIOLs over ACIOLs as they are associated with similar rates of coreal oedema, glaucoma and cystoid macular oedema as open-loop ACIOLs [21]. Gimbel et al. suggest that ACIOLs may not be well tolerated in the eyes with uveitis as they may exacerbate the associated disease, compromising the anatomy and function of the anterior segment. In one eye with iritis, implantation of ACIOL resulted in severe reaction and final vision was counting fingers [3,10]. In contrast, whilst our numbers are small, in our case series, all three patients who received ACIOLs had stable uveitis status at the final review, and all had vision better than their vision prior to their initial cataract surgery and better than their vision post-dislocation.

## Conclusions

In this retrospective series of five patients with uveitis and late in-bag IOL dislocation, the dislocation occurred on average 81 months after the cataract surgery. Patients had a reasonable visual recovery post-removal of dislocated IOL with or without secondary ACIOL placement. Further investigation is warranted to ascertain the strategies to prevent late IOL dislocation in uveitis patients.

## Consent

Written informed consent was obtained from the patient for the publication of this report and any accompanying images.

## Abbreviations

ACIOL(s): anterior chamber intraocular lens(es)
IOL(s): intraocular lens(es)
IVTA: intravitreal triamcinolone
PCIOL(s): posterior chamber intraocular lens(es)
AC: anterior chamber
ACIOL: anterior chamber intraocular lens
CNV: choroidal neovascularisation
HIV: human immunodeficiency virus
IOL: intraocular lens
IVTA: intravitreal injection of triamcinolone
LE: left eye
PCIOL: posterior chamber intraocular lens
PKE: phacoemulsification
Postop: postoperatively
Preop: preoperatively
RE: right eye
VA: visual acuity
VAR: visual acuity right eye
